# Promotional Properties of ACC Deaminase-Producing Bacterial Strain DY1-3 and Its Enhancement of Maize Resistance to Salt and Drought Stresses

**DOI:** 10.3390/microorganisms11112654

**Published:** 2023-10-28

**Authors:** Ye Yuan, Yanlei Shi, Zhenzhen Liu, Yonghong Fan, Min Liu, Mengkedala Ningjing, Yifei Li

**Affiliations:** National Demonstration Center for Experimental Biology Education, Xinjiang Key Laboratory of Biological Resources and Genetic Engineering, College of Life Science & Technology, Xinjiang University, Urumqi 830017, Chinalzz@stu.xju.edu.cn (Z.L.);

**Keywords:** ACC deaminase, abiotic stress, maize, plant growth-promoting rhizobacteria

## Abstract

Salt stress and drought stress can decrease the growth and productivity of agricultural crops. Plant growth-promoting bacteria (PGPB) may protect and promote plant growth at abiotic stress. The aim of this study was to search for bacterial strains that can help crops resist rises in drought and salt stresses, to improve crop seed resistance under drought and salt stresses, and to investigate the effect of bacterial strains that can help crop resist external stresses under different stress conditions. *Pseudomonas* DY1-3, a strain from the soil under the glacier moss community of Tien Shan No. 1, was selected to investigate its growth-promoting effects. Previous studies have shown that this strain is capable of producing ACC (1-aminocyclopropane-1-carboxylic acid) deaminase. In this experiment, multifunctional biochemical test assays were evaluated to determine their potential as PGPB and their bacterial growth-promoting properties and stress-resistant effects on maize plants were verified through seed germination experiments and pot experiments. The results showed that strain DY1-3 has good salt and drought tolerance, as well as the ability to melt phosphorus, fix nitrogen, and produce iron carriers, IAA, EPS, and other pro-biomasses. This study on the growth-promoting effects of the DY1-3 bacterial strain on maize seeds revealed that the germination rate, primary root length, germ length, number of root meristems, and vigor index of the maize seeds were increased after soaking them in bacterial solution under no-stress, drought-stress, and salt-stress environments. In the potting experiments, seedlings in the experimental group inoculated with DY1-3 showed increased stem thicknesses, primary root length, numbers of root meristems, and plant height compared to control seedlings using sterile water. In the study on the physiological properties of the plants related to resistance to stress, the SOD, POD, CAT, and chlorophyll contents of the seedlings in the experimental group, to which the DY1-3 strain was applied, were higher than those of the control group of seedlings to which the bacterial solution was not applied. The addition of the bacterial solution reduced the content of MDA in the experimental group seedlings, which indicated that DY1-3 could positively affect the promotion of maize seedlings and seeds against abiotic stress. In this study, it was concluded that strain DY1-3 is a valuable strain for application, which can produce a variety of pro-biotic substances to promote plant growth in stress-free environments or to help plants resist abiotic stresses. In addition to this, the strain itself has good salt and drought tolerance, making it an option to help crops grown in saline soils to withstand abiotic stresses, and a promising candidate for future application in agricultural biofertilizers.

## 1. Introduction

Soil salinization on a global scale is characterized by a combination of localized slowdowns and global intensification [1]. In the context of global warming, natural and anthropogenic factors and other conditions have accelerated the evolution of salinization and made it more sensitive [2]. Problems in the use of water resources in arid zones have led to secondary salinization, and salinization has become important within the framework of global change research [3]. Maize, as a cash crop, is susceptible to saline and alkaline land [4]. To meet the people’s needs and continue to improve their living standards and dietary structure change, the comprehensive utilization of maize has been rapidly developed. Research on its development and utilization has received increasing attention, and how to better breed maize in saline and dry land has become one of the hotspots of modern research [5].

High-salt- and drought-stress environments promote an increase in the concentration of ethylene in plants, and high concentrations of ethylene can lead to deleterious reactions in plants, such as leaf abscission, the loss of chlorophyll pigments, the inhibition of rooting and nodulation, and the promotion of leaf senescence [6,7]. Chandwani et al. noted that plants employ several strategies to improve growth and productivity in stressful environments, including the use of 1-aminocyclopropane-1-carboxylate acid(ACC) deaminase for bacterial production [8]. ACC is a precursor substance for ethylene synthesis, and ACC deaminase can be utilized by plant roots to irreversibly hydrolyze ACC to ammonia and α-butyric acid, reducing the levels of ethylene in the plants, which, in turn, affects the plant’s physiological, growth, and developmental processes [9]. The ACC deaminase-producing strains identified by Meena et al. mediated salt tolerance in wheat through an ABA-dependent cascade and salt-responsive ion transport system [10]. Similar conclusions were reached in the experiments of El-Ballat et al.: ACC deaminating strain inoculation upregulated the expression of genes related to stress tolerance [11]. In recent years, there has been more evidence that ACC can biosynthesize signaling functions alone to aid plant growth and combat various abiotic stresses. Plant growth-promoting rhizobacteria (PGPR) containing ACC deaminase are able to supply iron to the host plant, making them a potentially valuable fertilizer application [12]. At the same time, ACC deaminase-producing strains also show other probiotic properties, such as in the study by Lu et al. and Gao et al., which found that the secretion of IAA and phospholipids and the production of extracellular polysaccharides could enhance the nutrient uptake and antioxidant capacity of the plant and thereby maintain its balance of metal ions [13,14]. Many experiments have shown that the application of ACC deaminase-producing strains to the inter-root of plants has a positive effect on plant growth and physiology, but most of the experiments have focused on crops such as wheat and tomato, and few experiments have investigated the application of ACC deaminase-producing strains to the inter-root of maize [15,16,17,18,19]. To summarize, Phyto-producing bacteria with ACC deaminase activity assisted by several other mechanisms to help host plants become resistant to environmental stresses [20], increase crop yields, and regulate ethylene levels are emerging as a sustainable agricultural production strategy to reduce the effects of abiotic stresses [21].

To the best of our knowledge, there is a lack of data on the effect of ACC deaminase-producing PGPR on mitigating abiotic stress in maize cultivation in saline and dry lands. In arid and semi-arid farming areas, planting maize or rotating it with other crops is one of the effective ways to repair ecological damage and curb soil degradation. In order to select bacteria suitable for the soil environment of the region, the selection should take into account their adaptations, such as drought and salt tolerance, in addition to their own ability to produce pro-biomass. In this experiment, in order to meet the needs of crop cultivation in saline and dry land, targeted selection of drought-resistant, saline-resistant and other stress-resistant strains were inoculated to increase their inoculation effect and to ensure the effective play of the potential of bacterial promotion of plant growth, so as to give full play to the resource advantages of ACC deaminase-producing bacteria and to promote the sustainable development of agriculture. In this study, we initially investigated the effect of ACC deaminase-producing PGPR on the growth of maize, intending to lay a foundation for the efficient utilization of ACC deaminase-producing inter-root probiotics in the development of agriculture and the restoration of the environment, as well as to provide a scientific basis for the application of microbial fungicides in salinized areas.

## 2. Materials and Methods

### 2.1. Test Strains and Soil

The test strain was *Pseudomonas*. The test strain DY1-3 was isolated in the laboratory from the D sample zone of the soil under the glacial moss cover of Tianshan 1, and the maximum ACC deaminase activity was measured to be 4.734 ± 0.037 U/mg [22]. The maize variety used in the experiment was ‘Sweet Glutinous Jade 788’, and the soil used was maize-arable soil collected from the 103 Mission (Xinjiang, China).

Rhizospheric soil characterization: available nitrogen—36.33 mg/kg; pH—8.2; soil organic matter—15.69 g/kg; available phosphorus—16.78 mg/kg; available potassium—228.00 mg/kg; total content of water-soluble salt—6.54 g/kg; total nitrogen—0.94 g/kg.

### 2.2. Experiments on the Characterization of PGPR

The following experiments were carried out to investigate the growth and tolerance of the strain DY1-3: After determining the period of their development, all subsequent strains used were exponential-growth-phase strains. We characterized the ability of DY1-3 to produce plant growth-promoting substances using different media and reagents, and we determined its efficiency in producing plant growth-promoting substances.

To measure the effect of different incubation times on the growth of the strains, we added the bacterial suspension of the strains into the sterilized tryptic soy broth (TSB) liquid medium at 2% by volume. We incubated them at 30 °C and 180 rpm with oscillation and measured their growth every 2 h for 24 h. The development of the strains was determined via the absorbance turbidimetry method, using sterile water as a control. It was measured at OD600, and three parallel groups were set up for each set of experiments.

To assess the effects of salt stress and drought stress on the survival and proliferation of the strains, we inoculated the exponential-growth-phase strains obtained from the above experiments at an inoculum size of 200 μL per group into the TSB liquid medium with increasing NaCl concentrations (0, 2, 4, 6, 8, and 10%) and the TSB liquid medium with increasing Polyethylene glycol-6000 (PEG-6000) concentrations (0, 5, 10, 15, and 20%). Measurement was performed via absorbance turbidimetry at OD600 after incubation for 24 h after the inoculation of the strains, and three parallel groups were set up for each set of experiments.

**Figure 1:**
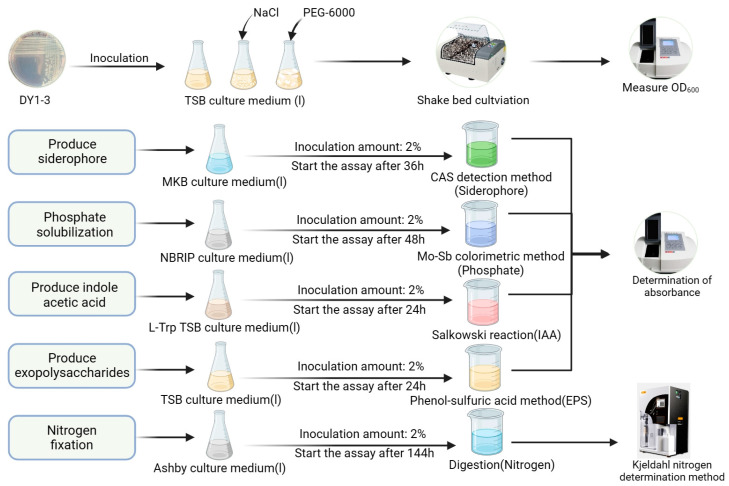
Experimental procedures for characterizing bacteria. DY1-3 was inoculated into TSB medium containing different concentrations of NaCl and PEG-6000 and standard TSB medium and incubated on a shaker for 24 h, during which the OD600 value of the culture solution was measured every 2 h to determine the ability of the strain to tolerate salinity and drought and to draw the growth curve of the strain. DY1-3 was inoculated with a selected solid medium to observe whether the strain had iron carrier, phosphorus solubilization, and nitrogen fixation characteristics, and it was inoculated with a selected liquid medium to cultivate and carry out the relevant detection reactions. Then, the absorbance of the reaction solution was measured with a spectrophotometer and compared with the standard curve to quantitatively determine the strain’s iron carrier, phosphorus-solubilizing, EPS-producing, and IAA-producing abilities, and the strain’s ability to fix nitrogen was measured via the Kjeldahl nitrogen fixation method.

In follow-up experiments, we measured the ability of the strains to produce various substances capable of promoting plant growth. We used the colorimetric method to determine the indole-3-acetic acid (IAA) synthesis ability of the strain. After determining the ability of the strain to produce IAA, the probiotic energy production was measured every 12 h at OD530 [23]. To identify the ability of the strain to produce iron carriers, the strain was inoculated into a solid culture covered with chromeazurol-S (CAS) on modified King’s B (MKB) and incubated at 28 °C and 180 rpm for 7 d [24]. If an orange halo was produced around the colony, then it could produce iron carriers. The bacterial solution was mixed with the CAS assay solution and then homogenized and left to stand for 1 h at 630 nm to determine the absorbance value (A) and the control of deionized water with a CAS assay (Ar). The A/Ar indicated the strain’s production of ferredoxin synthesis; the smaller the value of the A/Ar, the higher the ferredoxin synthesis production [25]. We characterized the strains for their ability to produce extracellular polysaccharides and determined their EPS-producing efficiency using the phenol–sulfuric acid method [26]. Pietro’s method was used to determine whether the strains were phosphorus-fusing [27], and the molybdenum antimony resistance colorimetric method was used to measure the amount of phosphorus fusing of the strains. In the nitrogen-fixing experiments [28], Ashby’s medium was used to determine whether the strains were capable of absorbing nitrogen, and the Kjeldahl method was subsequently used to determine the amount of nitrogen fixing of the strains [29], the specific experimental operation is shown in Figure 1.

### 2.3. Experiments with Maize Seeds Inoculated with Bacterial Strains

#### 2.3.1. Strain and Seed Treatment

The strain was inoculated into TSB medium, cultured to the plateau stage, centrifuged at 8000× *g* rpm for 10 min, and then an appropriate amount of sterile water was added to wash the bacterial impurities, repeated thrice. The bacterial suspension was made with sterile water. The concentration of the bacterial suspension was expressed by the value measured under the OD600, and the concentration gradient was based on the value of the OD600.

Distilled water was used to rinse the dust on the surfaces of the seeds several times. They were then sterilized twice with 75% fixed ethanol for 1 min each time, then soaked in NaClO for 5 min, and finally rinsed with sterile water 3–5 times. The thoroughness of the sterilization of the surfaces of the seeds was verified by using the flush of the last coating on the beef paste peptone medium. The prepared seeds were placed in different concentration gradients of bacterial suspensions for immersion.

**Figure 2:**
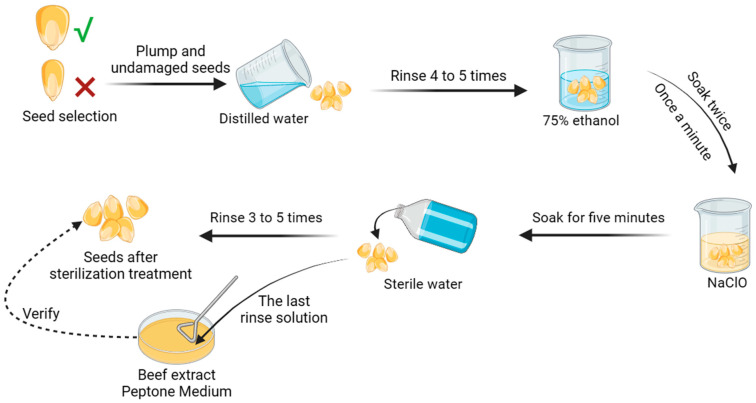
Corn seed treatment process. Corn seeds (marked with green √) with unbroken and full surfaces were selected and washed in distilled water, soaked in ethanol, soaked in sodium hypochlorite, and rinsed in sterile water in four steps to obtain seeds with complete bacterial elimination. The last rinsing solution was spread on beef paste plates to observe whether there were bacterial colonies to verify the complete sterilization of the seed surfaces.

Seeds soaked in sterile water were used as a negative control. The soaked seeds were laid flat on a sterile glass garden with different treatment solutions (4 layers of gauze and 1 layer of filter paper on the glass garden, with the filter paper upwards). Three replicates were used for each treatment group, and 20 full-grown maize seeds were used for each treatment group. The maize seeds were grown under greenhouse conditions with a light intensity of 4000 Lux, a light duration of 16 h/d, and a temperature of 30 °C for 10 d. The relative germination percentage, relative shoot length, relative primary root length, relative root meristem number, and relative vigor index were determined and calculated at harvest [30], the specific experimental operation is shown in Figure 2.

#### 2.3.2. Effect of Application on Seed Growth during Germination and Resistance to Salt and Drought Stresses

**Figure 3:**
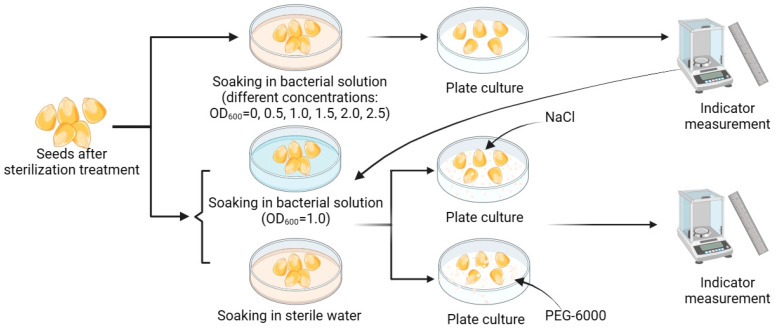
Experimental procedure for corn seed germination. The sterilized seeds were soaked in different concentrations of bacterial solution for 24 h and then incubated in flat dishes. Their growth indexes were measured after 10 days to select the bacterial-solution concentration with the best promotion of seed germination. The sterilized corn seeds were soaked in the optimum concentration of bacterial solution for 24 h. The control group was soaked in sterile water and incubated in Petri dishes with NaCl and PEG-6000; the stress solution was replenished every two days, and the indexes were measured after 10 days.

To measure the effects of different concentrations of bacterial suspensions on the maize seeds, we added bacterial suspensions with OD600 values of 0, 0.5, 1, 1.5, 2, and 2.5 to a sterile glass garden of cultured maize and, after obtaining the bacterial-suspension concentration with the optimal growth-promoting effect, we used this concentration for subsequent experiments. We applied the bacterial solution with the optimal growth-promoting concentration to the maize glass garden of cultured maize with NaCl concentrations of 0, 100, 200, 300, 400, and 500 mmol/L, versus the maize glass garden of cultivated maize with the addition of PEG-6000 concentrations of 0, 5, 10, 15, 20, and 25%, to validate that the bacterial strains helped the maize seeds to withstand the effects of different stresses, the specific experimental operation is shown in Figure 3.

### 2.4. Potting Experiments with Bacterial Solutions Applied to Corn

**Figure 4:**
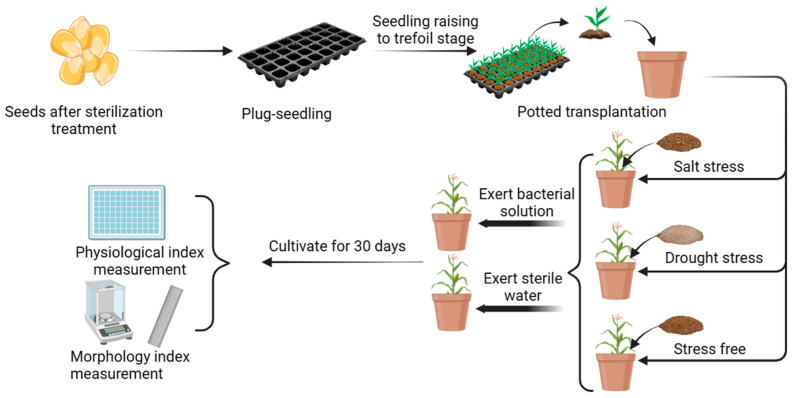
Corn-potting experimental procedure. After the antimicrobial treatment of corn seeds in the cavity tray, the seedlings in the three-leaf stage were transplanted into pots. After their growth was stable, the pots were divided into groups of applied salt stress, drought stress, and no stress, and the bacterial solution was applied to each group. The control group was administered sterile water. During this period, the stress solution was replenished every two days, and the bacterial solution was applied every three days. The morphological and physiological indexes were measured after thirty days of incubation.

Corn-cultivated soil was mixed with vermiculite in a 3:1 ratio and sterilized at 121 °C for 20 min. The salinity value of the soil was adjusted to the range of mildly salt-stressed soil by saturating the ground with 100 mmol/L salt solution. Corn-cultivated soils were dried and adjusted to the mildly drought-stress soil range. The treated seeds were sown into cavity trays and grown under a light intensity of 4000 Lux, a light duration of 16 h/d of light, and a temperature of 30 °C until the three-leaf stage, and they were then moved into the treated soil. The group with sterile water applied to each stress condition was the negative control, the no-stress group was set up as the experimental control group, and three groups were set up in parallel in each group of experiments. The measurements of the salinity and water content were carried out every day to ensure that the soil stresses were applied. After 30 d of growth, the relative plant height, relative stem thickness, relative root length, and relative root meristem number were measured with a kit (The test kit were purchased from Suzhou Grace Biotechnology Co., Ltd. (Suzhou, China)), and the relative plant height, relative stem thickness, relative root length, relative root meristem number, and chlorophyll, peroxidase (POD), catalase (CAT), superoxide dismutase (SOD), and malondialdehyde (MDA) contents were determined. The specific experimental operation is shown in Figure 4.

### 2.5. Statistical Data Analysis

The results are presented as mean ± SE (standard error) and were treated using analysis of variance (ANOVA) and Tukey’s honest significant difference test with a significance value of 5%. To determine the interaction between the factors tested, a multivariate analysis of variance (MANOVA) was performed using SPSS v. 23 software (IBM, Armonk, NY, USA). Lower numbers indicate significant differences between treatments at the *p* ≤ 0.05 level. The lowest numbers indicate highly significant difference between treatments at the *p* ≤ 0.01 level. The statistical charts were realized with GraphPad^®^ Prism v9.0 (GraphPad Software, San Diego, CA, USA).

## 3. Result

### 3.1. Potential of Bacterial Strains as Plant Growth Promoters

**Figure 5:**
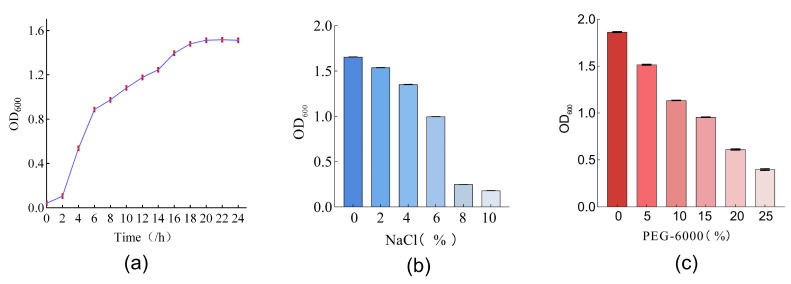
Growth and tolerance of strain DY1-3: (**a**) growth of strain DY1-3 at different incubation times; (**b**) growth of strain DY1-3 in media with different salt concentrations; (**c**) growth of strain DY1-3 in media with different PEG-6000 concentrations.

By observing the growth curves of DY1-3, we learned that the strain grew slowly at 0~2 h, multiplied in large quantities into the logarithmic phase at 2~16 h, and grew slowly after 18 h. The growth of the strain reached the plateau phase, and the subsequent experiments were carried out using the logarithmic-phase strains (Figure 5a). After incubating the strain in different salt concentration media for 24 h, the growth concentration of the DY1-3 in the media with 2%, 4%, and 6% salt concentrations decreased compared to that of the bacteria in the 0% salt concentration medium; the lowest concentration of the bacteria was observed when they were incubated in media with salt concentrations of 8% and 10%, with the OD600 values decreasing to 0.247 ± 0.002 and 0.180 ± 0.003, respectively (Figure 5b). After the injection of DY1-3 with the drought-stress medium containing different concentrations of PRG-6000 simulations, the bacterium showed a decreasing trend with increasing drought stress. Still, strain DY1-3 was more well adapted to the drought stress compared to its growth under salt concentration stress (Figure 5c). The above results showed that the optimal period for the inoculation of DY1-3 was between 2 and 6 h of incubation, and that the bacterium was well adapted to drought and salt stress and could be used for subsequent experiments.

**Figure 6:**
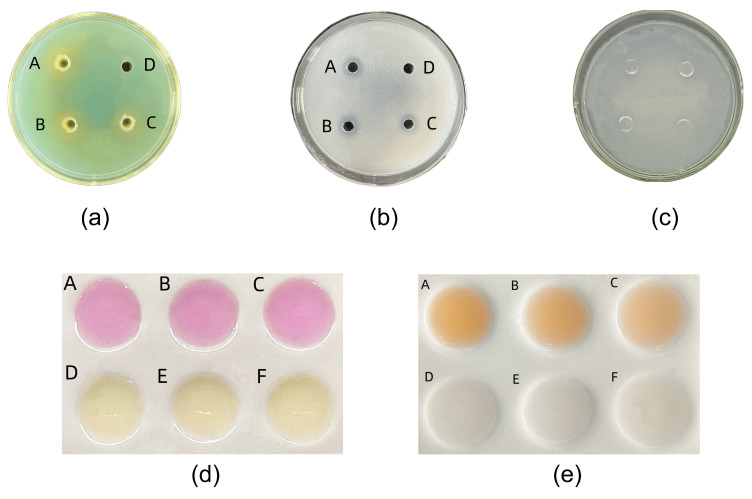
(**a**) Ability of strain DY1-3 to produce pro-biomass; (**b**) results of qualitative determination of IAA production by the strain; (**c**) results of qualitative determination of EPS production by the strain; (**d**) results of qualitative determination of iron carrier production by the strain; (**e**) results of qualitative determination of phosphorus-solubilizing capacity of the strain.

**Figure 7:**
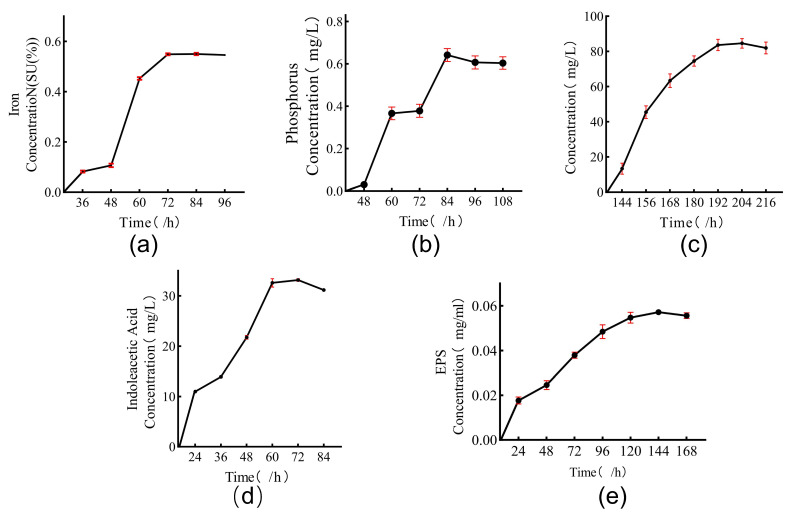
Efficiency of strain DY1-3 in producing pro-biomass at different fermentation times: (**a**) efficiency of IAA production by the strain; (**b**) efficiency of EPS production by the strain; (**c**) efficiency of iron carrier production by the strain; (**d**) efficiency of phosphorus solubilization by the strain; (**e**) efficiency of nitrogen fixation by the strain.

Through qualitative and quantitative experiments on the five growth-promoting properties of the strain, we obtained the following results: the strain showed a positive response to all five of our selected growth-promoting abilities (Figure 6). In the study on the efficiency of the strain to produce pro-biomass, the strain began to synthesize iron carriers, produce EPS and IAA, and solubilize phosphate in large quantities at 48 h. At 72 h, the strain synthesized iron carriers with a maximum SU of 54.55 ± 0.002%; at 60 h, the amount of synthesized IAA reached a peak of 32.590 ± 0.933 mg/L; at 144 h, the amount of EPS production reached a peaked at 0.05713 ± 0.000839 mg/mL; at 84 h, the amount of dissolved phosphorus reached a maximum value of 0.641 ± 0.035 mg/L. The strain started to undergo a large amount of nitrogen fixation after 144 h of growth, and it peaked at 83.777 ± 3.667 mg/L at 204 h. The strain also synthesized IAA at 60 h, and the amount of EPS produced at 144 h peaked at 32.590 ± 0.933 mg/L (Figure 7). In summary, the DY1-3 strain selected in this chapter had the ability to produce IAA, synthesize iron carriers, solubilize phosphate, and produce EPS in the above studies, and it can reproduce under higher salt stress and drought stress.

### 3.2. Effect of Adding Strain DY1-3 to Corn Seeds

**Figure 8:**
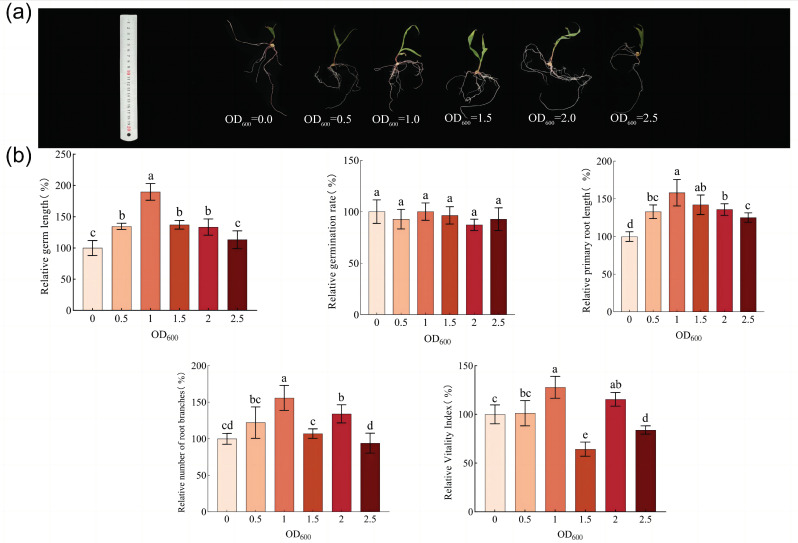
Effect of soaking maize seeds with different solution concentrations pf fungal solution on the growth of the maize seeds. (**a**) Representative photographs of maize seeds grown for ten days under different concentrations of fungal-solution treatments. (**b**) Bar chart of maize seed growth evaluation. Significant differences were analyzed using one-way ANOVA and t-test. Different letters indicate significant differences (*p* < 0.05).

By soaking corn seeds with different concentrations of bacterial solutions, we found (Figure 8) that there was no significant effect of the DY1-3 bacterial solution on the relative germination of the corn seeds at OD600 values of 0.0, 0.5, 1.0, 1.5, 2.0, and 2.5 (*p* > 0.05). The best promotion effects on the relative germ length, relative primary root length, relative root meristem number, and relative number of maize seeds were achieved when the concentration of the bacterial solution was an OD600 value of 1.0. The group with OD600 = 1 had a significant increase (*p* < 0.05) compared with the other experimental groups. The relative germ length of the maize seeds was 189.73 ± 12.57% of the control group without the bacterial solution, the relative primary root length was 158.11 ± 19.97%, and the close root meristem number was 155.80 ± 21.02%. The relative vigor index was 127.85 ± 13.17% of the control group (158.11 ± 19.97%). The relative root meristem number was 155.80 ± 21.02% of the control group, and the relative vigor index was 127.85 ± 13.17% of the control group. In summary, a certain concentration of DY1-3 bacterial solution has obvious promotional effects on corn seed germination in terms of the relative germ length, the relative length of the main root, the relative number of root branches, and the vitality index, and an OD600 value of 1.0 is when the promotional effects are the most obvious. Thus, the subsequent selection of an OD600 value of 1.0 bacterial solution was used to carry out the research on the bacterial strain’s effect on the plant’s ability to promote its growth and improve its resistance.

**Figure 9:**
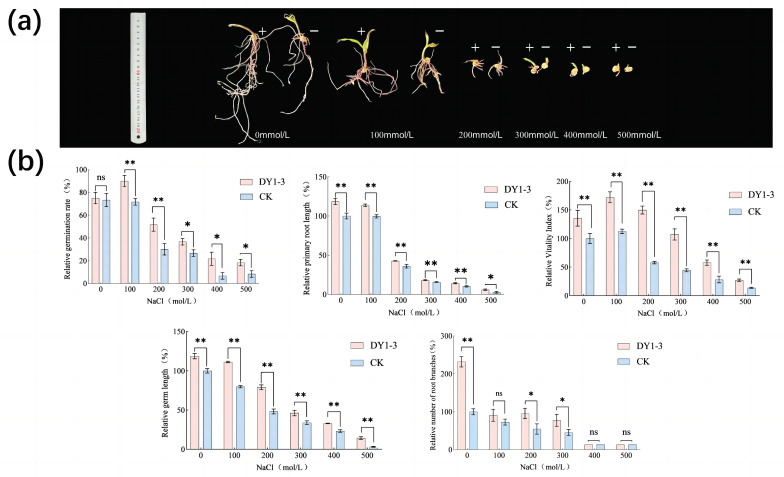
Effect of soaking seeds in bacterial solution at different salt concentrations on the growth of maize seeds. (**a**) Representative photographs of maize seeds grown at different salt concentrations for ten days + indicates that maize seeds were subjected to bacterial solution; − indicates that maize seeds were soaked in sterile water. (**b**) Bar graph of corn seed growth evaluation. Significant differences were analyzed using one-way ANOVA and t-test. ns: not significant; *: significant (*p* < 0.05); **: highly significant (*p* < 0.01). CK stands for the growth process without the addition of the bacterial strain DY1-3 cultured in sterile water only.

In the experiment on the ability of the strain to help corn seeds resist salt stress (Figure 9), compared with the control group in which the seeds were soaked in sterile water, the experimental group in which the bacterial solution was applied had a highly significant effect on the germination rate of the plants under salt stress with salt concentrations of 1 mmol/L~200 mmol/L (*p* < 0.01). The germination rate of the experimental group increased by 1.21-fold, 1.26-fold, and 1.72-fold compared with that of the control group. The germination rate of the mushroom solution-soaked group under salt stress at salt concentrations of 300 mmol/L~500 mmol/L was significantly higher than that of the control group without the application of the bacterial solution, being 1.38, 3.25, and 2.2 times higher than that of the control group, respectively (*p* < 0.05). Still, the bacterial solution did not contribute significantly to the relative germination rate of the seeds in the absence of stress (*p* > 0.05). The bacterial-solution-soaked seeds significantly increased the relative germ length of the maize seeds (*p* < 0.01), with 0.19-fold, 0.40-fold, 0.64-fold, 0.37-fold, 0.41-fold, and 3.36-fold increases in the experimental group compared to the control group, respectively. Compared to the control group with no bacterial-solution-soaked seeds, the relative root length of the primary root of the seeds under the salt stress of 0 mmol/L~400 mmol/L had highly significant increases of 0.19-fold, 0.14-fold, 0.19-fold, 0.15-fold, and 0.38-fold, in that order (*p* < 0.01). Under 500 mmol/L salt stress, the seeds’ relative primary root length increased significantly by 1.29-fold compared with the control after soaking the seeds in the bacterial solution (*p* < 0.05). The bacterial-solution soaking of the maize seeds resulted in a highly significant 1.32-fold increase in the relative root meristem number of maize seeds (*p* < 0.01), which did not show the significance of the experimental group. However, the relative root meristem number of the experimental group was 1.25-fold higher than that of the treated group at a salt stress of 100 mmol/L. At a salt stress of 200 mmol/L~300 mmol/L, the relative root meristem number of the bacterial-solution-soaked seed group was significantly higher than that of the experimental group (*p* < 0.05), being 1.75 times versus 1.70 times higher than that of the experimental group. There was no significant difference in the relative root meristem numbers between the experimental and control groups under the salt-stress treatments of 400 mmol/L~500 mm/L (*p* > 0.05). The use of the DY1-3 bacterial solution for soaking maize seeds had different effects on the relative vigor index of the maize seeds as a function of the salt concentration. At NaCl concentrations of 100 mmoL/L and 200 mmoL/L, the relative vigor index of the maize seeds increased from 137.52 ± 5.14% at 0 mmoL/L to 176.28 ± 4.17% and 154.79 ± 5.27%, respectively. With the increase in the salt concentration, the inhibition of the vigor index of the maize seeds was strengthened, and its relative vigor index continued to decline, compared with the relative vigor index of the maize seeds treated with sterile water. The treatment of maize seeds using the DY1-3 bacterial solution had a highly significant promoting effect (*p* < 0.01). In summary, strain DY1-3, as a class of plant inter-root-promoting bacteria, can promote plant growth and improve the plant’s resistance to salt stress by increasing the relative germination rate and relative vigor index of corn seed germination and the relative germ length, relative radicle length, and relative number of root branches of corn seeds.

**Figure 10:**
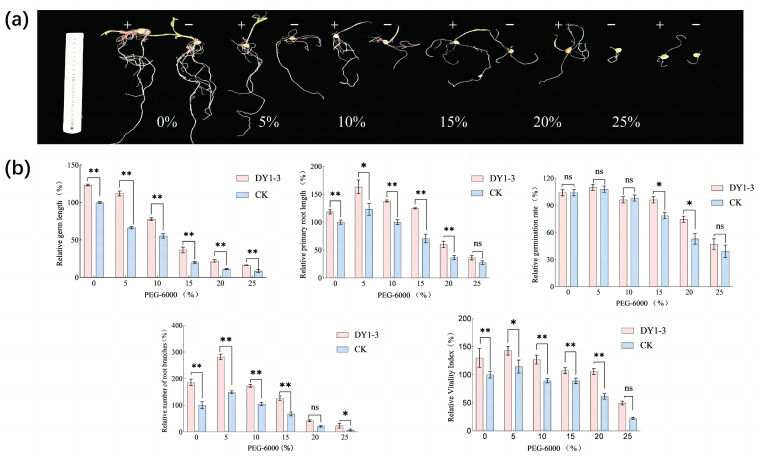
Effect of soaking maize seeds in bacterial solution at different PEG-6000 concentrations on their growth. (**a**) Representative photographs of maize seeds grown at different PEG-6000 concentrations for ten days + indicates that the maize seeds were subjected to bacterial solution; − indicates the maize seeds were soaked in sterile water. (**b**) Bar graph of corn seed growth evaluation. Significant differences were analyzed using one-way ANOVA and t-test. ns: not significant; *: significant (*p* < 0.05); **: highly significant (*p* < 0.01). CK stands for the growth process without the addition of the bacterial strain DY1-3 cultured in sterile water only.

Strain DY1-3 helped the maize seeds to better withstand drought stress (Figure 10). Compared to the control group of seeds soaked in sterile water, the seeds soaked in the bacterial solution under drought stress of PEG-6000 (15~20%) had a significant effect on the germination percentage of the plants (*p* < 0.05), which was 1.23, 1.40, and 1.72 times higher than that of the control group. Soaking the seeds in the bacterial solution under drought stress (PEG-6000 concentration of 5–25%) did not significantly increase the germination percentage of the maize seeds (*p* > 0.05). Under different concentrations of drought stress, soaking the seeds in the bacterial solution had a highly significant effect on the relative germ length at different concentrations, which increased 1.23, 1.69, 1.40, 1.84, 1.98, and 1.88 times (*p* < 0.01), respectively, compared with the control. At a drought-stress PEG-6000 concentration of 25%, the effect of soaking the seeds in the bacterial solution on the primary root length was not significantly different from that of the control group (*p* > 0.05), and at a PEG-6000 concentration of 5%, soaking the seeds in the bacterial solution significantly increased their primary root length (*p* < 0.05), which was 1.32 times longer than that of the experimental group. At PEG-6000 concentrations of 10–20%, soaking the seeds in the bacterial solution increased the relative primary root length of the experimental group significantly (*p* < 0.01), and the relative primary root length of the comparison group was increased by 0.38, 0.76, and 0.64 times, respectively. The relative root meristem number of the experimental group was significantly increased at PEG-6000 concentrations of 5~20% (*p* < 0.05), which was 1.89, 1.64, 1.86, and 2.00 times higher than that of the control group, respectively. When the drought stress PEG-6000 concentration was 25%, the bacterial-solution-soaked seeds significantly increased the relative root meristem number of the corn seeds (*p* < 0.05), which was 3.3 times higher than that of the control group. The relative vigor index of the maize seeds increased from 128.43 ± 8.14% at 0% stress to 143.28 ± 4.29% at 5% PEG-6000 solution, and the treatment with the DY1-3 bacterial solution had a highly significant promotion effect in the range of 0–20% stress on the vigor index (*p* < 0.01); no significant promotion effect was observed at the 25% PEG-6000 solution (*p* > 0.05).

### 3.3. Effect of Strain DY1-3 Access on Morphological Indicators of Maize Seedlings

**Figure 11:**
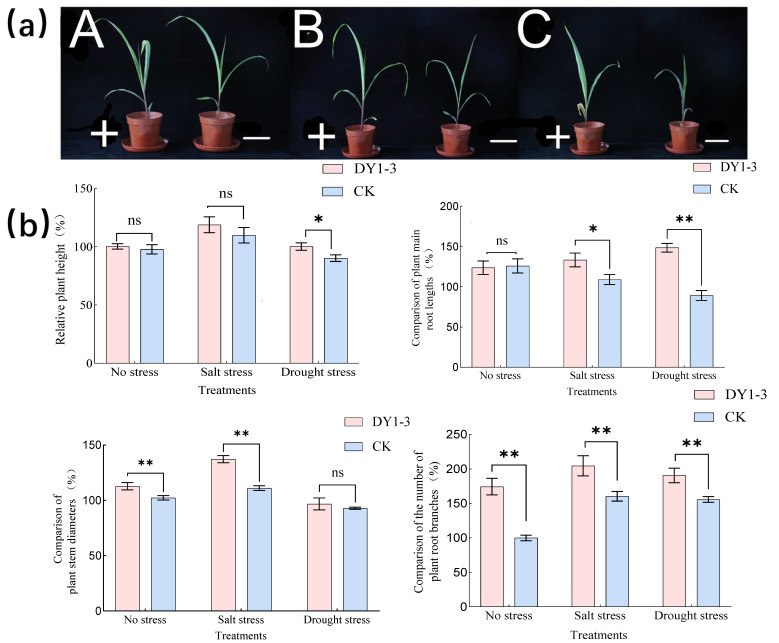
Effect of the DY1-3 bacterial strain on morphological indexes of maize seedlings grown under different stresses for 30 days. (**a**) Representative photographs of maize seedlings grown under different stresses for thirty days; + indicates that the maize seedlings were watered with DY1-3 bacterial solution during the growth period; − indicates that the maize seedlings were watered with sterile water during the growth period. A: The CK group did not add any stress to plants; B: The group that causes salt stress to plants; C: The Group that cause drought stress to plants. (**b**) Bar graph of maize seedling growth evaluation. Significant differences were analyzed via one-way ANOVA and t-test. ns: not significant; *: significant (*p* < 0.05); **: highly significant (*p* < 0.01). One-way ANOVA and t-test for significant difference analysis. ns: not significant; CK stands for the growth process without the addition of the bacterial strain DY1-3, cultured in sterile water only.

The addition of the DY1-3 bacterial solution significantly (*p* < 0.05) affected the resistance of the maize seedlings to stress (Figure 11). The relative plant height, stem thickness, primary root length, and root meristem number of the experimental group of the maize seedlings were 100.21 ± 2.68%, 112.72 ± 2.21%, 123.84 ± 9.33%, and 174.42 ± 6.98%, respectively, in the no-stress environment. The fungal-solution application had a highly significant effect on the stem thickness and the number of root meristems of the maize seedlings (*p* < 0.01). The relative plant height, stem thickness, primary root length, and root meristem number of the experimental group of maize seedlings under salt stress were 118.76 ± 5.16%, 137.21 ± 3.46%, 133.51 ± 9.67%, and 204.65 ± 16.28%, respectively. The application of the bacterial solution had a highly significant effect on the stem thickness and root meristem number of the maize seedlings under the salt-stress environment (*p* < 0.05), which were 1.25 and 1.29 times higher than those of the group without the bacterial solution application, respectively. The application of the bacterial solution significantly increased the relative primary root length of the maize seedlings (*p* < 0.05), which was 1.16 times higher than that of the group without the bacterial addition. However, the application of the bacterial solution did not significantly affect the relative plant height of the corn seedlings under salt stress (*p* > 0.05). Under drought stress, the relative plant height, stem thickness, primary root length, and root meristem number of the experimental group of maize seedlings were 100.14 ± 2.46%, 96.82 ± 3.87%, 148.71 ± 3.11%, and 190.70 ± 9.30%, respectively. The application of the bacterial solution had a highly significant effect on the number of root meristems and the primary root length of the maize seedlings under drought stress (*p* < 0.01), which were 1.26 times and 1.58 times higher those of the control group, and significantly increased the relative plant height by 1.13 times compared with the control group. The application of the bacterial solution had a significant effect on the number of roots and primary root length of the maize seedlings that survived under drought stress (*p* < 0.05) (1.26 and 1.58 times those of the control group, respectively), and significantly increased the relative plant height of the maize seedlings by 1.13 times that of the control group. The application of the bacterial solution did not significantly affect the stem thickness of the maize seedlings surviving under drought stress (*p* < 0.05).

### 3.4. Effect of Strain DY1-3 Access on Physiological Indexes of Maize Seedlings

**Figure 12:**
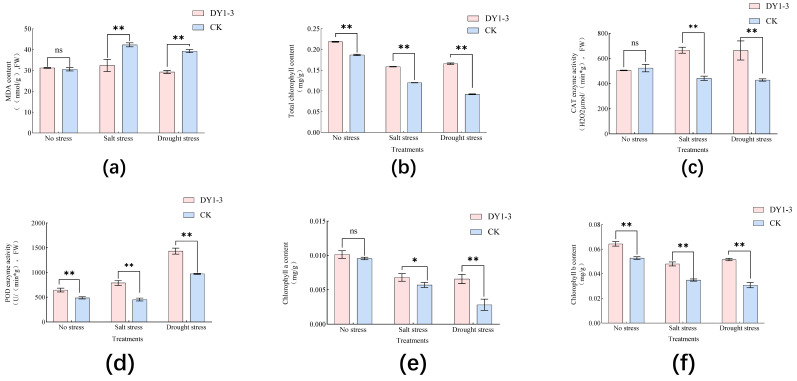
Effects of DY1-3 strain on physiological indexes of maize seedlings grown under different stresses for 30 days. (**a**) MDA activity of maize seedlings under different stresses. (**b**) SOD activity of maize seedlings under different stresses. (**c**) CAT activity of maize seedlings under different stresses. (**d**) POD activity of maize seedlings under different stresses. (**e**) Chlorophyll a content of maize seedlings under different stresses. (**f**) Chlorophyll b content of maize seedlings under different stresses. Significant differences were analyzed via one-way ANOVA and *t*-test. ns: not significant; *: significant (*p* < 0.05); **: highly significant (*p* < 0.01). CK stands for the growth process without the addition of the bacterial strain DY1-3, cultured in sterile water only.

The application of the DY1-3 bacterial solution also had a significant effect on the physiological indexes of the maize seedlings (*p* < 0.05) (Figure 12). The contents of CAT, MDA, POD, SOD, chlorophyll a, and chlorophyll b in the experimental group of maize seedlings in the stress-free environment were (504.61 ± 2.36) μmol/(min·g), (31.22 ± 0.01) nmol/g, (644.33 ± 33.21) U/(min·g), (0.87 ± 0.02) U/g, (0.010 ± 0.00061) mg/g, and (0.064 ± 0.0019) mg/g. The addition of the bacterial solution did not significantly affect the CAT, MDA, or chlorophyll b in the maize seedlings in the stress-free environment (*p* > 0.05); however, the application of the bacterial solution resulted in a significant increase in the chlorophyll content of the maize seedlings, which was 1.07 times higher than that of the control (*p* < 0.05). The application of the bacterial solution had a highly significant effect on the POD, SOD, and chlorophyll b contents of the maize seedlings (*p* < 0.01), which were compared with the control. They were increased by 0.38, 1.07, and 0.95 times, respectively, compared with the control.

The CAT, MDA, POD, SOD, chlorophyll a, and chlorophyll b contents in the experimental group of maize seedlings under the salt-stress environment were (665.57 ± 15.10) μmol/(min·g), (32.41 ± 3.34) nmol/g, (788.33 ± 31.33) U/(min·g), (0.70 ± 0.02) U/g, (0.0068 ± 0.00013) mg/g, and (0.048 ± 0.0013) mg/g, respectively. Under salt stress, the application of the bacterial solution significantly elevated the CAT (*p* < 0.01), POD, SOD, chlorophyll b, and total chlorophyll contents of the maize seedlings by 1.39-fold, 1.48-fold, 2.30-fold, 1.31-fold, and 1.32-fold compared to the control, respectively, and significantly reduced the MDA activity in the experimental group to 81% of that of the control (*p* < 0.01). Under salt stress, applying the bacterial solution significantly increased the chlorophyll content to 1.36 times that of the control (*p* < 0.05).

Under the drought-stress environment, the CAT, MDA, POD, SOD, chlorophyll a, and chlorophyll b contents in the experimental group of maize seedlings were (663.67 ± 33.96) μmol/(min·g), (29.29 ± 0.54) nmol/g, (1432 ± 62.21) U/(min·g), (0.66 ± 0.01) U/g, (0.0066 ± 0.00027) mg/g, and (0.051 ± 0.0012) mg/g, respectively. The application of the bacterial solution significantly increased the CAT, POD, SOD, chlorophyll a, and chlorophyll b contents of the experimental group by raising them to 1.61, 0.72, 1.178, 3.09, and 1.59 times those of the experimental group (*p* < 0.05), respectively. The MDA value of the experimental group was significantly reduced to 72% of that of the control group (*p* < 0.01).

## 4. Discussion

PGPR is a group of beneficial bacteria present in the soil or attached to plant roots that promote plant growth, uptake, and utilization of nutrients and can significantly promote plant growth when subjected to environmental stresses [31,32] (*p* < 0.05). Under high-intensity salt and drought stress, the large amount of ethylene produced by plants can adversely affect various processes, such as fruit ripening, flowering, and root formation, thereby hindering plant growth. ACC deaminase is not only able to inhibit the increase in the ethylene concentration in high-stress environments, but it is also able to individually biosynthesize signaling functions, regulate plant growth, and participate in cell wall signaling, the defense of cell division, and resistance to pathogen virulence [33]. The strain used in this study was the bacterium *Pseudomonas*, which was isolated from the soil under the moss community from the No.1 glacier of Tianshan Mountain and cultured in the laboratory. To better carry out the subsequent utilization of the bacterial strain, this study explored its growth and its tolerance to salt and drought stresses, and we found that it was able to reproduce and develop at higher salt and drought concentrations. This excellent tolerance lays the foundation for its subsequent application as a plant bioconcentration agent in salty and arid lands. Strain DY1-3 has superior ACC deaminase production and salt and drought tolerance compared to the existing ACC deaminase-producing strains in our laboratory and some ACC deaminase-producing strains found in other laboratories [34,35,36]. It has been shown that ACC deaminase is limited by abiotic stresses to function better under stressful conditions [37,38,39,40]; however, from the experiments on the growth of the maize seeds at different concentrations of the bacterial solution in the present study and the subsequent comparison between the added-bacteria group and the non-added-bacteria group in pots under stress-free conditions, it was evident that the administration of the bacterial solution significantly (*p* < 0.05) improved the growth of the maize seedlings and seeds. This indicates that the bacterium can produce not only ACC deaminase, which is a plant growth-promoting strain, but also other probiotic substances.

Strain DY1-3 has IAA production, iron phagocytosis, phosphorus solubilization, EPS production, and nitrogen fixation abilities, a variety of growth-promoting abilities to not only use ACC deaminase to help plants resist stress in adverse environments, but also in the absence of stress environments to promote plant growth. In the study of Khan et al. strains with ACC deaminase have the ability to induce systemic resistance to production of exo-polysaccharide, siderophore, ammonia, IAA, and efficiently solubilized zinc and phosphate under in vitro conditions [41]. The nitrogen-fixing ability of plants helps them absorb nitrogen from the atmosphere [42,43,44]. To help plants that do not have the ability to convert or uptake nitrogen themselves [45], strain DY1-3 is more capable of nitrogen fixation than other ACC deaminase-producing strains [46,47,48] and can better provide nitrogen nutrients for plant growth, thereby significantly reducing the use of artificial nitrogen fertilizers and alleviating soil environmental pollution (*p* < 0.05). About 95% of the soil’s phosphorus is ineffective, which plants can not directly absorb. DY1-3 can dissolve phosphorus and improve the nitrogen fixation capacity of the soil so that the iron and zinc elements in the soil can be fully utilized [49,50]. The results of Chaveevan et al. also indicate that ACC deaminase-producing strains are phosphorus-producing [51]. Not only can the presence of DY1-3 convert the ineffective phosphorus into a form that can be absorbed and used by the plant to promote the growth of plant roots, but the production of IAA can also be useful in the decomposition of nutrients and the metabolism, transportation, storage, and protection of cells [52], and through the stimulation of the growth of the roots, to cope with the role of the external environment on the plants under stress. It was also demonstrated by Zhang S et al. that strains with ACC deaminase capacity can help plants resist salt stress and promote plant growth by secreting IAA [53]. In the study of Vurukonda SS, strains with ACC deaminase activity could help plants to reduce the effects of drought environment while also producing EPS to help them to resist abiotic stresses [54]. The EPS produced by DY1-3 enhances the plant stress resistance to the environment and helps root colonization [55]. Only a minimal amount of iron (Fe^3+^) can be absorbed by organisms in nature, and the iron carrier produced by DY1-3 is a small-molecule organic substance produced by microorganisms in iron-deficient environments that enhances iron uptake. In addition, the producers of iron carriers and chelating iron can adsorb other heavy metals, such as lead, arsenic, aluminum, magnesium, zinc, copper, cobalt, and strontium [56]. Kumar, A et al. also found an iron carrier producing ability in bacteria with ACC deaminase [57]. Strain DY1-3 can produce a variety of biomass-promoting substances, and this study not only characterized whether it could make various types of growth substances, but it also investigated the timing of its biomass production, which lays a foundation for the subsequent application of biomass-producing strains.

In this study on the effect of different bacterial-solution concentrations on seed germination, we found that the application of the DY1-3 bacterial solution did not promote the germination rate of the seeds in the stress-free environment. This is because the germination rate of maize seeds under stress-free conditions is only the strongest correlation among the quality of their internal storage material, water content, etc., and the addition of bacterial strains does not change some of the characteristics of maize seeds themselves [58]. However, the addition of the bacterial solution significantly (*p* < 0.05) increased the relative root meristem number, primary root length, shoot length, and vigor index of the maize seeds compared to the control group, which may be attributed to the positive effect of growth hormones and other substances produced by the bacterial strains on the growth and development of the plants. In the environment with a NaCl concentration of 0–100 mmol/L, strain DY1-3 helped the maize plants to resist the stress effects mainly by increasing the shoot length, primary root length, and seed vigor of the maize seeds. The increase in the vigor index of the maize seeds at this stage also indicated that the addition of strain DY1-3 enhanced the rate of uptake of the storage material by the maize seeds [59]. When salt stress with a salt concentration of 100 mmol/L was applied, the germination rate of the maize seeds assisted by strain DY1-3 was higher than that of the unstressed soil, which may be due to the role of ACC deaminase produced by strain DY1-3 under this stress, and which promotes the cell division of maize seeds to accelerate their uptake of stored materials, increasing the germination rate [60]. The addition of strain DY1-3 significantly increased the germ length (*p* < 0.05), primary root length, root branching number, and relative vigor index of the maize seeds when a salt stress of 200 mmol/L–300 mmol/L was applied. The good promotional effect on all the indexes at this concentration may be due to the fact that this type of salt concentration enables the average growth of DY1-3 to produce growth substances, such as IAA and other growth substances, and, at the same time, the ACC deaminase acts synergistically with the plant, which helps to protect it against the adverse effects of the stress [61]. The increase in the relative number of root meristems of the maize seeds by the bacterial solution at ambient NaCl concentrations greater than 400 mmol/L is not significant (*p* > 0.05), and this is because, at the seed germination stage, high-salt-stress conditions affect plant growth regulators, slowing down their physiological processes and inhibiting the dispersal of seed roots [62]; thus, maize seeds reduce the distribution of the number of root meristems to maintain adequate water and the rational use of nutrients but lengthen their main roots to better search for water [63,64,65]. The synergistic effect of strain DY1-3 with the maize seeds also followed this feature, which significantly increased the primary root length of the maize seeds compared to the control group at a salt concentration of 400–500 mmol/L (*p* < 0.05), and the addition of the bacterial solution resulted in a significant increase in the shoot length and vigor index of the maize seeds compared to the unaddressed group (*p* < 0.05). The findings of Atif A et al. showed similar results to those of the present study: inoculation of the halotolerant bacteria PM22 reduced the severity of salinity stress in plants and increased root and shoot length at various salt concentrations (0, 180, 240, and 300 mM) [66]. Mahmood N et al. also showed that inoculation of inter-plant root-promoting bacteria containing ACC-deaminase may be an effective way to improve maize growth and yield under salt stress conditions [67]. At the time of applying drought stress to the treated seeds and pressure of PEG-6000 at a concentration of 5–10%, Because corn is more sensitive to salt stress than drought stress at the seed stage, low drought stress has less of an effect on the germination rate of corn seeds [68]; thus, applying the bacterial solution at this stage did not significantly improve the germination rate of the corn seeds (*p* > 0.05). In terms of different drought-stress conditions, unlike salt stress, the relative root meristem number of the maize seeds was significantly increased in the high-drought-stress environment (PEG-6000 concentration of 20–25%) after they were soaked in the bacterial solution (*p* < 0.05) [69]. This was due to the fact that the cell division of the maize seeds was accelerated under high drought stress with the help of the bacterial strain DY1-3, and the process accelerated the rapid growth of their roots and shoots. The development of shoots ensures the seed’s intake of water from the air [70,71], and the increase in the number of root meristems allows for the absorption of more water in a smaller area to support the plant’s survival and development [72]. However, the change in the length of the primary root of the maize seeds under high drought stress was not significantly correlated with the presence or absence of the bacterial solution when soaking the seeds, which might also be related to the fact that drought stress inhibits the growth of the plant (*p* > 0.05) [73]. Murali M et al. also showed that the incorporation of bacteria with ACC deaminase enhances seed adaptation to drought stress [74]. This is corroborated by the study of Zahir Z A et al. who concluded that inoculation with bacteria containing ACC deaminase could be helpful in eliminating the inhibitory effects of drought stress on the growth of seeds [75].

In order to increase the generalizability and reproducibility of the experiment, the stress indicators chosen for the potting experiment were mildly saline and mildly arid soils, with the largest proportion of saline and arid soils [76,77,78]. The results of the study showed that when no stress was applied, the suitable environment did not induce the strain to produce substances such as ACC deaminase-like enzymes that aid in plant growth [79], and other biomasses produced by the DY1-3 strain alone did not have significant growth effects on the relative plant height or primary root length of the maize plant (*p* < 0.05); however, the application of DY1-3 in our results still significantly increased the stem thickness and the number of root meristems of the maize plants to increase the nutrient uptake and transport by the maize seedlings (*p* < 0.05). The addition of DY1-3 significantly increased the contents of SOD, POD, and chlorophyll in the plants under stress-free conditions (*p* < 0.05). Still, it did not have a significant effect on the contents of MDA or CAT (*p* < 0.05), which may be due to the fact that IAA and other plant growth-promoting substances secreted by the colonization of strain DY1-3 need to be decomposed and utilized by the plants through the production of POD and other substances, and that DY1-3 can enhance plant growth and development through the increase in the contents of chlorophyll and SOD to promote the plant’s cell division and the enhancement of the photosynthesis of the nutrients produced by the plant. The addition of DY1-3 significantly increased the SOD (*p* < 0.05), POD, and chlorophyll contents in the plants under stress-free conditions but did not have a significant effect on the MDA and CAT contents, which may be due to the fact that IAA and other plant growth-promoting substances secreted by the colonization of strain DY1-3 need to be catabolized and utilized by the plants through the production of POD and other substances [80], and the addition of DY1-3 significantly enhanced the plant growth, but did not have a significant effect on the MDA or CAT contents (*p* < 0.05). DY1-3 can promote cell division and enhance photosynthesis to produce nutrients to strengthen plant growth and development by increasing the contents of chlorophyll and SOD [81,82]. Plants in stress-free environments that are not harmed by external abiotic stresses do not produce large amounts of MDA or CAT, which are substances that need to be accumulated by the plants after they receive stress [83,84]. Under low salt and drought stress, the addition of strain DY1-3 significantly increased the values of the root meristem number and root length of the maize plants, promoted the secretion of SOD, POD, CAT, chlorophyll a, and chlorophyll b (*p* < 0.05), and reduced the content of MDA in the maize seedlings. The results of Vijayrao Bomle et al. were similar to those of the present study, PGPR with ACC deaminase activity was used as a green bio-inoculant reducing the impact of saline conditions [85]; Baber Ali et al. also showed the same opinion as in the present study that inoculation with ACC deaminase active strains can reduce oxidative stress markers in maize under salt stress [86]. In this study, under the effect of adversity stress, strain DY1-3 significantly improved the antioxidant effect of the maize plants and better helped them to scavenge the adverse effects of free radicals, reduce the greening reaction caused under high-stress environments, and enhance photosynthesis under adversity (*p* < 0.01) [87,88], which significantly helped them to resist the damage caused by abiotic stresses (*p* < 0.01).

The bacterium *Pseudomonas* DY1-3 used in this experiment has various plant growth-promoting functions. It can help plants resist damage, including that caused by drought and salt stresses, making it a promising candidate for future agricultural applications. More experiments will be conducted to investigate its mechanism of action and application range to study its protective effects on plants under pressure and lay the foundation for future field applications.

## 5. Conclusions

Soil salinization and the expansion of arid zones are significant constraints to the development of agricultural productivity. The strain DY1-3 used in this study significantly improved the growth and development of maize seeds and seedlings under stress conditions, based on which the following conclusions were drawn:

1. The results of this study show that DY1-3 has a strong ability to survive in high-salinity and high-drought environments, and is capable of producing iron carriers, dissolving phosphorus, synthesizing IAA, producing EPS, and nitrogen fixation.
2. In the absence of abiotic stress, the addition of bacterial solution did not significantly increase the germination rate of maize seeds, but under drought and salt stress conditions, the use of bacterial solution of DY1-3 strain to soak maize seeds significantly increased the germination rate of maize seeds as well as increased the germination length of the seeds, the number of root meristems, and the vitality index, etc., and mitigated the inhibitory effects of salt and drought stress on the growth of maize seeds.
3. The application of strain DY1-3 had no significant effect on the growth of the crop under stress-free conditions, but in the study of the physiological characteristics of the crop related to stress resistance, the contents of SOD, POD, CAT, and chlorophyll were significantly higher than those of the control seedlings without the application of the bacterial broth, and the incorporation of the bacterial broth significantly lowered the content of MDA in the experimental group of seedlings.

This study only explored the effect of a single bacterium on the growth-promoting and stress-resistant effects on plants, and in the future, it can be compounded with other microorganisms with stress-resistant and growth-promoting functions to be used as microbial agents to improve soil fertility, nutrient uptake, and stress-resistant properties in plants, thus increasing the yields and quality of agricultural crops. In conclusion, the use of inter-root biotrophic bacteria such as strain DY1-3 in combination with appropriate organic fertilizers will contribute to crop cultivation in arid zones and salinized lands as a proper mitigation measure for the sustainability of agricultural systems in the context of the increasing warming of the climate and salinization of soils in the future.

## Data Availability

The data presented in this study are available upon request from the corresponding authors.
